# Items

**Published:** 1871-06

**Authors:** 


### Items.

Chloral Hydrate in Nocturnal Enuresis—Dr. J. B. Bradbury states that he has used the chloral hydrate successfully in the treatment of this affection. He mentions one case particularly, a girl fifteen years of age, who had wetted her bed every night for nine years. She was ordered fifteen grains of the chloral every night, and after taking the first dose there was no return of the complaint. She had no relapse at the end of six weeks. Other cases are given showing the marked beneficial results from the use of the agent in this affection. He claims chloral possesses the following advantages over belladonna: 1. The effect of belladonna is not so immediate, frequently taking weeks to produce any marked control over the disease; whereas the influence of chloral hydrate is most rapid, the malady frequently disappearing after the first dose of the remedy. This quick improvement can not be over-estimated in the treatment of these affections, upon which the mind exerts a powerful influence. 2. That belladonna some times induces profuse diarrhea. 3. That belladonna, when pushed to the extent to which it is neccessary to be really efficacious, not unfrequently impairs vision, etc., which is not the case with chloral hydrate. 4. That belladonna has often failed to be of any service.—*Brtish Medical Journal.*

The Replanting of Teeth.—Dentists are now testing a plan proposed by Mr. Coleman, an English dentist, as follows: Extract the tooth, clear away caries and the contents of the pulp cavities and canals, wash out with carbolic acid, fill the canals with cotton dipped in carbolic acid, fill the cavity, scrape off all diseased periosteum and cementum, leaving the healthy portions of the mucous membrane attached to the neck of the tooth; bathe alveolus and the tooth in a solution of carbolic acid, and return the tooth to its socket. Out of fourteen cases Mr. Coleman succeeded with nine; operating on bicuspids and molars.—*Pacific Med. and Surg. Journal.*

Women are now allowed to attend the cliniques at the Pennsylvania hospital, in Philadelphia. The result is that the number of hospital tickets sold is not half as great as formerly. The audience at the lectures, moreover, consists almost entirely of Homœopaths, Eclectics, and the like, to the great disgust of the lecturers. As these gentlemen give their services gratuitously, their wishes ought certainly to be respected.

The *Medical Record* gives an account of an interesting case of intussusception cured by the persistent injection of salt and water. The introduction of air was abondoned after a second trial, on account of the pain it caused.

